# Is Anyone Ready to Save a Life? An Examination of Cardiac Emergency Preparedness in Schools

**DOI:** 10.1111/josh.13517

**Published:** 2024-10-20

**Authors:** Heather K. Baker

**Affiliations:** ^1^ School of Education Aurora University Aurora IL

**Keywords:** CPR, AED, CPR training, cardiac emergency preparedness, schools

## Abstract

**BACKGROUND:**

This study explored the cardiac emergency preparedness of school employees in Illinois, as well as their attitudes toward cardiopulmonary resuscitation (CPR) and automated external defibrillator (AED) training.

**METHODS:**

One thousand two hundred seventy‐six school employees completed an online survey regarding their school's cardiac emergency preparedness, as well as their access to CPR/AED training, confidence and willingness to perform CPR/AED, and attitudes toward CPR policies and mandates.

**RESULTS:**

In total, results from 1273 school employees were analyzed. School employees in Illinois are not prepared to respond to a cardiac emergency, but desire training, plans, and drills. Demographic analyses revealed statistically significant differences in cardiac emergency preparedness between individuals of different personal and school characteristics.

**CONCLUSIONS:**

School employees in Illinois are not prepared to respond to a cardiac emergency. To increase preparedness for cardiac emergencies at school, schools should implement CPR/AED training for all employees, cardiac emergency response plans, and cardiac emergency response drills.

**IMPLICATIONS FOR SCHOOL HEALTH POLICY, PRACTICE, AND EQUITY:**

Policies should be implemented at the state and local level to support cardiac emergency preparedness in schools, including mandated CPR/AED training for all school employees, cardiac emergency response plans for every building, and required cardiac emergency response drills.

Sudden cardiac arrest (SCA) is a public health crisis that affects people of all ages. In the United States, there are more than 356,000 out‐of‐hospital cardiac arrests each year, with a survival rate of approximately 10%.^1^ During SCA, time is of the essence, as victims who do not receive cardiopulmonary resuscitation (CPR) and a shock from an automated external defibrillator (AED) from a bystander within the first 3 to 5 minutes of collapse have little to no chance of survival.^1^, ^2^

On average, an estimated 20% of the adult and child population enters a school each day, and life‐threatening emergencies, such as SCA, can and do occur within the school setting, to both children and adults.^3^ Currently, SCA is the leading cause of death for student‐athletes, accounting for more than half of all student deaths, and an estimated 1 in 70 high schools have a SCA on campus each year.^4^, ^5^, ^6^, ^7^ In the event of SCA, immediate intervention is essential, and survival rates are significantly higher in schools where the school supplies the AED itself, rather than waiting for emergency medical services, and in schools with a written and practiced plan versus schools without.^7^ In order for victims to have the best odds of survival, schools must have a planned and practiced response, including a system for communication during an emergency, the creation and practice of a cardiac emergency response plan (CERP), an AED on campus, and CPR/AED training for all school employees.^3^

In an effort to utilize schools as a gateway for CPR/AED training large numbers of individuals, the American Heart Association (AHA) and International Liaison Committee on Resuscitation formally endorsed CPR training in schools in 2004.^8^ As a result of this recommendation, most states now require CPR training for high school students, while few states have implemented mandated CERPs or CPR/AED training for school employees.

In Illinois, several laws pertain to cardiac emergency preparedness. Colleen O'Sullivan's Law, which requires fitness facilities to have an AED on site and school gyms to have AEDs nearby, was passed in 2005 after Colleen O'Sullivan passed away at a fitness facility where an AED was unavailable for use.^9^ Although this law requires public schools in Illinois to purchase and place AEDs in close proximity to the school gymnasium, this law does not require school employees to be educated on how or when to use the AED.

In 2014, Lauren's Law was passed after the tragic death of high school senior Lauren Laman, a student at St. Charles North High School in St. Charles, Illinois.^10^ After experiencing SCA during a school‐sponsored dance practice, Lauren Laman passed away after her coaches and fellow bystanders failed to provide CPR and defibrillation from a nearby AED.^10^ As a result of her passing, 105 Illinois Compiled Statutes (ILCS) 110/3 mandates that CPR and AED training appear within the health curriculum for high school students.^11^ While high school health teachers may be more likely to be trained due to their obligation to teach CPR/AED to students, the current statute allows school employees to teach CPR/AED to students without CPR/AED certification themselves. As written, this mandate intends to ensure that all high school students are trained in CPR/AED, but does not require the training of any school employees. This mandate leaves adults in school buildings responsible for training students, but largely unprepared for cardiac emergencies. Although this mandate has appeared on the Illinois State Board of Education's mandated training list since 2014, compliance with and implementation of this law is undocumented.^12^

Due to the historical lack of CPR/AED training requirements for school employees in Illinois, this descriptive study aimed to measure the cardiac emergency preparedness of PK‐12 school employees in Illinois and gauge their interest in becoming CPR/AED trained. This study also examined the inequities associated with access to CPR/AED training and the cardiac emergency preparedness of school employees.

## METHODS

### Participants/Procedures

A quantitative descriptive study was conducted among school employees working in Illinois public and private schools, with students in grades Preschool through 12th grade. In March of 2021, a participation invitation email was sent to 6399 individuals who were members of the Illinois Principals Association in the 2019‐2020 or 2020‐2021 school year. The email sent to administrators invited individuals to take the survey and asked them to forward the provided participation invitation email and survey link to all employees in their building. Participation in the study was optional, and survey dissemination to school employees was dependent upon the principal's willingness to forward the survey. The survey link remained active for 6 weeks prior to being closed for final data analysis.

### Instrumentation

This descriptive study used a survey design to examine an overview of the cardiac emergency preparedness of PK‐12 public, private, and charter school employees in Illinois, as well as school employees' attitudes toward and willingness/desire to participate in CPR/AED training. In addition, this study examined the role of potential moderating variables, including role, years of experience, age, race/ethnicity, gender, school region type, grade levels served, school type, school socioeconomic status (SES), and school size. A survey instrument was constructed with Qualtrics Survey Software containing a combination of 5‐point Likert‐scale questions, multiple‐answer multiple‐choice categorical questions, single‐answer categorical questions, and demographic information questions. Survey questions were presented in 5 sections: demographics/background information (52 questions), willingness to participate/desire to participate in training (17 questions), confidence to perform CPR/AED (1 question), willingness to perform CPR/AED (13 questions), and knowledge of CPR/AED skills (12 questions). A sixth set of 13 questions was presented only to individuals who answered that they had attended training at school, and included follow‐up questions regarding the quality and content of the school‐provided class.

In the informed consent form, participants were made aware that no personally identifiable data was collected, and that participants could skip questions or exit the survey at any time.

### Data Analysis

IBM SPSS Statistics 25.0 was used for all data analysis. Basic descriptive statistics were applied to each research question, and likelihood ratio chi‐square tests were conducted separately for each demographic and district characteristic to determine if any significant relationships were found. Kruskal‐Wallis tests were performed for all Likert‐scale responses. Wilcoxon rank‐sum tests were performed to compare Likert responses between demographic and school characteristic groups. All statistical tests were performed at the .05 significance level.

## RESULTS

### Characteristics of Participants

In total, 1276 public and private school employees in Illinois completed the survey. Data from 3 respondents who self‐identified as working outside of the state of Illinois was excluded from this study. The remaining 1273 respondents were PK‐12 public and private school employees in the state of Illinois, and were comprised primarily of 38.2% teachers, 33.0% administrators, and 28.8% other positions (Table 1).

**Table 1:** Respondent Characteristics by Number and Percentage of Respondents

Characteristic	Respondents
Race/ethnicity (N = 1239)	
American Indian or Native Alaskan	2 (0.2%)
Asian	7 (0.6%)
Black	30 (2.4%)
Hispanic or Latinx	41 (3.3%)
White	1146 (92.5%)
Two or more races	10 (0.8%)
Other	3 (0.2%)
Current role (N = 1273)	
Teacher	486 (38.2%)
Administrator	420 (33.0%)
Administrative assistant/secretary	42 (3.3%)
Paraprofessional/classroom aide	119 (9.3%)
Support staff (social worker, nurse, etc.)	138 (10.8%)
Custodian/maintenance	10 (0.8%)
Security personnel	6 (0.5%)
Food services	8 (0.6%)
Substitute teachers	16 (1.3%)
Coaches	50 (3.9%)
Club sponsors	19 (1.5%)
Other	31 (2.4%)

Participants were additionally asked to report on several school characteristics and demographics (Table 2). The majority of respondents indicated that they worked for public schools, served elementary students, and had a student enrollment of 0‐499 students. Most respondents also reported working at schools with high SES.

**Table 2:** School Characteristics by Number and Percentage of Respondents

Characteristic	Respondents
School region (N = 1240)	
Urban	121 (9.8%)
Suburban	662 (53.4%)
Rural	457 (36.8%)
School type (N = 1221)	
Public school	1169 (95.7%)
Non‐public school	52 (4.3%)
Socioeconomic status (N = 1218)	
Low SES	394 (32.3%)
High SES	419 (34.4%)
Moderate SES	242 (19.9%)
Unspecified	163 (13.4%)

### CPR/AED Training and Knowledge

Participants were asked a series of categorical questions related to their participation in CPR/AED training at school, as well as several multiple‐choice questions that measured their knowledge of basic CPR/AED skills. While most respondents indicated that they had provided first aid (85.9%) or responded to a life‐threatening emergency (49.1%) at school at least once in their career, only 26.5% of participants felt that their school had provided enough training to respond to SCA. Just over half of respondents (59.4%) indicated that they were offered CPR/AED training at school, and 65.8% of individuals offered training stated they had chosen to participate in the school‐offered training. Of the individuals who had participated in school training, 67.9% indicated that they had received training from their school within the last 1‐2 years.

Upon further analysis of demographic and school characteristic factors, statistically significant differences were found in the amount of training offered to individuals based on school size, SES, population, and employee race. Results indicated that individuals who worked at larger schools were more likely to have participated in training than employees of smaller‐sized schools ($\chi_{3}^{2}$ = 8.9, *P* = .03), and individuals who worked at high schools were more likely to have participated in training than non‐high school employees ($\chi_{1}^{2}$ = 3.8, *P* = .05). Individuals who worked in an urban setting ($\chi_{4}^{2}$ = 24.6, *P* < .001) as well as individuals who served more low‐income students ($\chi_{6}^{2}$ = 56.4, *P* < .001) were less likely to have been offered CPR/AED training at school. Non‐white employees not only reported lower levels of participation in school‐based CPR training ($\chi_{1}^{2}$ = 7.9, *P* = .005) but also reported being offered less training than their white peers ($\chi_{2}^{2}$ = 11.3, *P* = .004).

In this study, student access to mandated training was briefly explored. Currently, Illinois schools are mandated to teach CPR/AED as a part of the high school health curriculum.^10^, ^11^, ^12^ However, the results of this survey indicated that implementation of this law may be uneven. Of all high school employees surveyed, only 44.4% of high school respondents reported that their school offered CPR/AED training to some or all students, and only 19.6% reported that their school required training for some or all students. The remaining respondents indicated that they were unaware of their school's student requirement for CPR/AED training, which could be due to their role within the school and amount of knowledge surrounding school curriculum.

**Figure 1:**
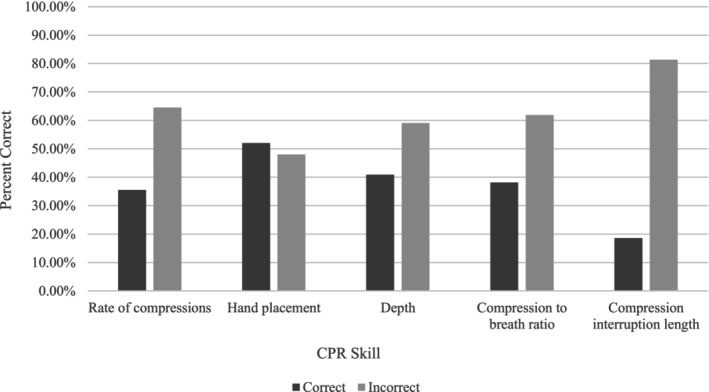
Summary of Knowledge of Basic CPR Skills

**Figure 2:**
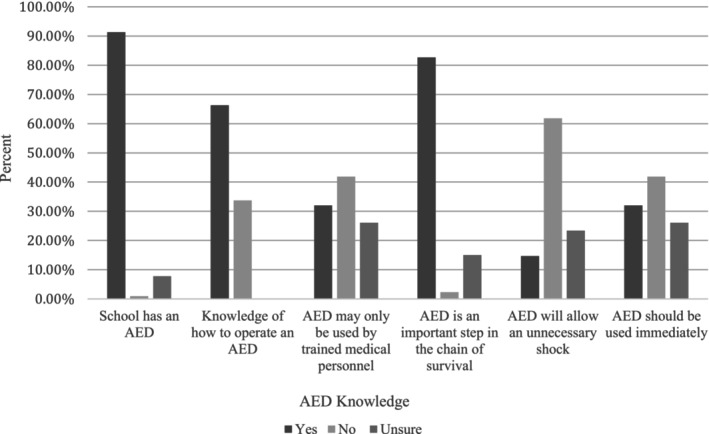
Summary of Responses for AED Placement and Knowledge

To further explore CPR/AED readiness, respondents were given a series of multiple‐choice questions to measure their knowledge of basic CPR/AED skills. As seen in Figure 1, the majority of respondents did not correctly answer basic CPR/AED skill‐based multiple‐choice answer questions, and most were unable to correctly identify the appropriate rate of compressions for CPR, compression depth, breath‐to‐compression ratio, or acceptable compression interruption length. Results also indicated that school employees lacked basic knowledge of how and when to use an AED (Figure 2). Of the participants in this study, only 32.0% knew that an AED should be used immediately when responding to SCA, and only 41.9% knew that an AED could be used by a layperson. Individuals working at schools with low to moderate SES performed more poorly on skill‐based questions, specifically, how to use an AED ($\chi_{3}^{2}$ = 30.4, *P* < .001) and proper rate of chest compressions ($\chi_{3}^{2}$ = 21.4, *P* < .001). Individuals working in an urban setting were also less likely to know how to use an AED than individuals working in schools with moderate to high income populations ($\chi_{2}^{2}$ = 7.4, *P* < .03).

Previous studies have shown that participation in CPR/AED training can increase preparedness to respond to a cardiac emergency.^8^, ^13^, ^14^, ^15^ Similarly, results of this survey indicated that school employees who participated in school‐provided CPR/AED training answered all skill‐based questions correctly at a higher rate than those who had not been trained (*P* = < .001 for all skills). However, CPR/AED trained individuals still struggled to correctly answer basic questions regarding CPR/AED. The majority of trained individuals could not correctly identify the proper rate of compression (57.9% incorrect), compression depth (50.1% incorrect), compression‐to‐breath ratio (54.2% incorrect), or compression interruption length (77.1% incorrect), suggesting that there may be problems with skill retention or quality of training. Only 38.8% of CPR/AED trained individuals knew that an AED should be utilized immediately on a victim of SCA, and only 69.3% of trained individuals reported knowing that an AED cannot administer an unnecessary shock.

### Attitudes Toward Performing CPR/AED

Participants were asked several 5‐point Likert scale questions to gauge their confidence and willingness to respond to a cardiac arrest at school, with a victim of any age. Only 39.5% of respondents reported feeling prepared to respond to a cardiac emergency (mean = 2.9, SD = 1.25). Although participants generally felt unprepared to respond to SCA, school training proved effective, as individuals who participated in school CPR/AED training (mean = 3.24) reported feeling significantly more prepared to respond to a cardiac emergency than individuals who had not participated in training (mean = 2.21, *P* < .001).

Inequities in feelings of preparedness were identified, primarily related to SES and school setting. Individuals from schools with a student population of high to moderate SES reported being significantly more prepared to respond to a cardiac emergency than individuals from low‐income schools (*P* = .004) and were more likely to report having a cardiac emergency response plan (*P* = .032). On the other hand, a higher number of individuals from low and unspecified SES (*P* = .005), as well as individuals working in an urban setting (*P* = .020), reported that they felt that their school did not provide enough training to respond to SCA. The difference in feelings of preparedness between high school and non‐high school employees was not statistically significant (*P* = .16).

Consistent with the self‐reported low levels of preparedness, less than half of respondents (32.5%, mean = 2.8, SD = 1.23) indicated that they were confident in their ability to recognize the signs of SCA, and less than half (48.7%, mean = 3.2, SD = 1.21) indicated that they were confident in their knowledge of what to do when someone collapses. However, despite low levels of skill‐based knowledge and confidence to identify SCA, over half of respondents indicated they were confident in their ability to perform CPR (56.2%, mean = 3.3, SD = 1.22), give high‐quality chest compressions (54%, mean = 3.3, SD = 1.24), or use an AED (50.5%, mean = 3.1, SD = 1.38). The majority of respondents were also very willing to provide CPR/AED to a victim, as 83.6% indicated they were willing to give hands‐only CPR (mean = 4.2, SD = .945), 61.1% were willing to give a victim mouth‐to‐mouth resuscitation (MMR) (mean = 3.6, SD = 1.14), and 74.5% reported being willing to use an AED (mean = 3.92, SD = 1.15).

Differences found between levels of confidence and willingness to perform CPR/AED and school SES, school region, and race were highly significant. Confidence levels of individuals from schools with low SES were significantly lower than individuals from moderate SES for all CPR/AED skills (*P* < .001 for all skills). School employees from urban (*P* = .032) and low SES schools (*P* < .001) reported being less willing to use an AED on a victim than school employees from suburban and higher SES schools. Additionally, participants from low SES schools reported being significantly less willing to give an individual hands‐only CPR than participants from schools with moderate to high SES (*P* = .011). Non‐White employees rated themselves as being less willing to perform CPR (*P* = .02) and AED (*P* = .03) than White employees.

Participation in CPR/AED training proved useful in improving willingness and confidence to perform CPR. Individuals who had participated in school CPR/AED training reported higher levels of confidence to use an AED (mean = 3.54) than individuals who had not received training at school (mean = 2.36, *P* < .001), and individuals who had participated in training also showed higher levels of confidence for performing CPR (mean = 3.61, *P* < .001), chest compressions (mean = 3.58, *P* < .001), and rescue breaths (mean = 3.45, *P* < .001). Individuals who had participated in CPR/AED training were more willing to perform hands‐only CPR (mean = 4.13, *P* < .001), MMR (mean = 3.73, *P* < .001), and use an AED (mean = 4.16, *P* < .001) than untrained participants. When asked about barriers to giving CPR/AED to a victim, lack of practice (44.3%) and lack of confidence in knowledge or skills (39.1%) were the 2 highest‐rated barriers to performing both CPR and AED.

### Cardiac Emergency Response Plans and Drills

The creation and distribution of a CERP, in combination with practicing cardiac emergency response drills, can help school employees prepare to respond to a cardiac emergency by offering the opportunity to discuss and apply skills learned in training courses.^3^ The majority of respondents (62.6%) indicated that their school either did not have a CERP or that they did not have knowledge of the steps in their school's plan. Additionally, the vast majority (74.6%) of respondents indicated that they had never participated in a cardiac emergency response drill at school, with 15.2% indicating that they were unsure if their school performed drills of this type.

### Attitudes Toward CPR/AED Training Requirements

**Figure 3:**
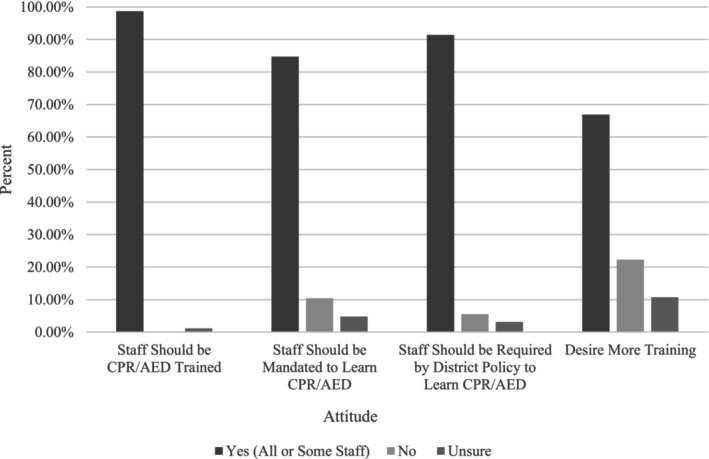
Summary of Responses for Employee Attitudes Toward Training/*Re*‐training

Although the majority of participants indicated that they were not prepared to respond to SCA, school employees were largely in support of measures to increase cardiac emergency preparedness. As shown in Figure 3, 97.4% of school employees indicated that they wanted their school to offer CPR/AED training and retraining, 98.7% believed that some or all school employees should be CPR/AED trained, and 84.7% believed that state law should mandate some or all school employees to learn CPR/AED at once in their career. The majority believed that their school should require training for some or all employees through local policy (91.4%). The vast majority of school employees (97.2%) believed that their school should have a CERP, and 84.1% believed that their school should complete a cardiac emergency response drill. Over half (66.9%) of employees reported wanting more training on how to recognize and respond to SCA at school, and 95.7% indicated they would attend a free CPR/AED class if offered by the school.

## DISCUSSION

Cardiac emergency preparedness in schools, including CPR/AED trained individuals and cardiac emergency plans and drills, is essential for increasing the odds of survival for victims of SCA.^3^, ^8^, ^13^, ^14^, ^15^, ^16^, ^17^ While CPR/AED training mandates in the United States have been largely focused on high school students, the focus should be broadened to include all school employees, to ensure that the entire school community knows how to respond effectively in the event of an emergency.^3^, ^13^, ^14^, ^15^, ^16^, ^17^, ^18^

The findings of this study indicate that while many school employees are willing to perform CPR/AED on a victim, most are not prepared for a cardiac emergency, as they do not possess strong knowledge of basic CPR/AED skills, and do not have plans or drills in place to prepare for such an emergency. In accordance with existing literature, participants in this study who had been CPR/AED trained were better prepared for a cardiac emergency, as they displayed higher levels of confidence to perform CPR/AED, reported being more willing to perform CPR/AED, indicated higher levels of feelings of preparedness, and had better knowledge of CPR/AED skills.^13^, ^14^, ^17^ Individuals from demographics who were offered less training reported being less willing and confident to initiate CPR/AED, and less prepared for a cardiac emergency. These findings, along with past research, support the need for CPR/AED training for school employees, as training increases the likelihood that people will not only be confident and willing to perform CPR/AED on a victim, but will also perform it well enough to increase the odds of survival.^13^, ^14^, ^17^

While individuals who had participated in CPR/AED training displayed higher levels of preparedness and knowledge, most trained individuals still incorrectly answered CPR/AED skill‐based questions and reported that they desired more training. Consistent with recommendations from the AHA, this finding implies that CPR/AED training on its own may not be enough to fully prepare an individual for a cardiac emergency, and that a CERP and cardiac emergency drill should also be utilized in preparing for cardiac emergencies at schools.^3^, ^18^ The quality of training being offered and retention of skills should be further explored.

## IMPLICATIONS FOR SCHOOL HEALTH POLICY, PRACTICE, AND EQUITY

The findings of this study have important implications for current school safety practices. Although not currently prepared for a cardiac emergency, most participants strongly supported increasing the cardiac emergency preparedness of schools by offering or requiring CPR/AED training regularly, performing cardiac emergency response drills, and having CERPs. Given the favorable attitudes of school employees toward cardiac emergency preparedness requirements and mandates, policies should be implemented at the state and local level that support cardiac emergency preparedness in schools, including mandated CPR/AED training for all school employees, CERPs for every building, and required cardiac emergency response drills. Schools in states with mandates already in place for student and/or employee training should ensure that this practice is being implemented, and resources need to be distributed equitably to schools to aid with compliance with training requirements.

On July 1, 2024, House Bill 5394 was signed into law in Illinois.^19^ This law amends the School Safety Drill Act (105 ILCS 128/5) and the Critical Health Problems and Comprehensive Health Education Program Act (105 ILCS 110/3) and requires each public and private school, beginning with the 2024‐2025 school year, to provide school employees with knowledge surrounding hands‐only CPR and AED, and to create a CERP. Other states should model legislation after this bill to take steps toward being more prepared for cardiac emergencies.

The results of this study also highlight the inequities across school populations for cardiac emergency preparedness. In general, individuals who worked in urban settings, schools with lower SES, and non‐white school employees were less prepared for cardiac emergencies, with less access to training. Organizations and individuals committed to increasing school safety must be aware of these disparities and ensure that resources are equitably distributed to ensure that all school employees across all demographics are prepared for cardiac emergencies.

With or without state mandates or board policies, school administrators, school boards, and school nurses can work collaboratively to adopt practices that fully support cardiac emergency preparedness in schools. Each school should annually offer CPR/AED training to employees, create a cardiac emergency response team and CERP, and run a cardiac emergency response drill, all of which can be implemented with little to no cost to a school.

### Resources and Support for Schools

There are several resources available to schools looking to become cardiac emergency prepared, and in offering or seeking CPR training versus certification, schools can avoid lengthy class time requirements and costs for certification cards and trainers. Local fire departments, police departments, and hospitals can often connect schools with CPR trainers and training materials for little to no cost. Schools can also consider the use of materials often found in schools, such as dodgeballs, to practice skills on if they lack CPR training manikins. CPR/AED training, instead of certification, can be done without a licensed instructor, utilizing free videos found online.

Multiple organizations offer free tools, templates, and resources for schools to use to prepare for cardiac emergencies. Project ADAM offers free materials that can be accessed online by schools looking to become cardiac emergency prepared.^20^ Through Project ADAM, schools can find free drill and CERP templates to create local materials quickly and easily and can follow a checklist to obtain an official Heart Safe School designation. Recently, the AHA has partnered with Project ADAM to make similar CERP templates and materials available on their website as well.^21^

Individuals and organizations working to improve outcomes for SCA victims should focus their efforts on providing resources to schools to allow them to provide more training and education regarding cardiac emergencies, as well as free and low‐cost options for CPR/AED training. Organizations should lobby for increased funding to support these initiatives in schools and should work to partner with schools to provide training and help with the creation of CERPs.

### Limitations

The participation invitation for this study was emailed to school administrators through an email list provided by the IPA, and the participation level of respondents was uncontrollable. This survey was only distributed to schools at which an administrator was an active member of the IPA in the 2019‐2020 or 2020‐2021 school year, which did not encompass every principal in the state of Illinois. Distribution of the survey to school employees was dependent on the principal's willingness to extend the survey to all employees in their building. Study respondents may have been biased toward more active engagement with CPR training and may have had more positive opinions regarding CPR/AED training than non‐respondents. Due to the mandate for CPR training for high school students in Illinois, there may be more cardiac emergency preparedness and awareness at the high school level. Based on an individual's role within the school setting, some participants may have been unaware of their school's practices regarding the CPR/AED training of employees or students.

### Conclusions

Overall, school employees are not prepared for a cardiac emergency, but desire more knowledge and are very willing to implement measures that would make them more prepared for SCA. With or without state mandates, schools should work to ensure that they offer CPR/AED training regularly to all employees, have a CERP, and perform cardiac emergency response drills at least once per year. Legislators and organizations interested in school health and safety should make cardiac emergency preparedness in schools a top priority and should not limit their training requirements to students alone.

### Human Subjects Approval Statement

IRB approval was obtained from Aurora University and included the provision that all participants could exit the survey at any time or skip any questions that they did not wish to answer.

### Conflict of Interest

No potential competing interest to report.
